# Ionotropic receptors at hippocampal mossy fibers: roles in axonal excitability, synaptic transmission, and plasticity

**DOI:** 10.3389/fncir.2012.00112

**Published:** 2013-01-09

**Authors:** Arnaud J. Ruiz, Dimitri M. Kullmann

**Affiliations:** ^1^Department of Pharmacology, UCL School of PharmacyLondon, UK; ^2^Department of Clinical and Experimental Epilepsy, UCL Institute of NeurologyLondon, UK

**Keywords:** 2-photon microscopy, GABA_A_ receptor, granule cell, immunogold, kainate receptor, mossy fiber bouton, NMDA receptor, single channel

## Abstract

Dentate granule cells process information from the enthorinal cortex en route to the hippocampus proper. These neurons have a very negative resting membrane potential and are relatively silent in the slice preparation. They are also subject to strong feed-forward inhibition. Their unmyelinated axon or mossy fiber ramifies extensively in the hilus and projects to stratum lucidum where it makes giant en-passant boutons with CA3 pyramidal neurons. There is compelling evidence that mossy fiber boutons express presynaptic GABA_A_ receptors, which are commonly found in granule cell dendrites. There is also suggestive evidence for the presence of other ionotropic receptors, including glycine, NMDA, and kainate receptors, in mossy fiber boutons. These presynaptic receptors have been proposed to lead to mossy fiber membrane depolarization. How this phenomenon alters the excitability of synaptic boutons, the shape of presynaptic action potentials, Ca^2+^ influx and neurotransmitter release has remained elusive, but high-resolution live imaging of individual varicosities and direct patch-clamp recordings have begun to shed light on these phenomena. Presynaptic GABA_A_ and kainate receptors have also been reported to facilitate the induction of long-term potentiation at mossy fiber—CA3 synapses. Although mossy fibers are highly specialized, some of the principles emerging at this connection may apply elsewhere in the CNS.

## Introduction

There are approximately one million granule cells within the rat dentate gyrus, all projecting a thin unmyelinated axon that forms a single parent fiber in stratum lucidum, where it makes synaptic contacts onto CA3 pyramidal cells and various types of interneurones (Amaral et al., 1990; Acsady et al., 1998). These unusual axons (or mossy fibers) provide one of the most powerful glutamatergic input in the brain amid the low basal firing rate observed in granule cells *in vivo* (<0.5 Hz) and the inability of granule cells to fire action potentials for extended periods of time (Jung and McNaughton, 1993). Mossy fiber—CA3 synapses express a unique form of frequency-dependent facilitation of transmitter release that has a pronounced effect with only modest increases in presynaptic firing frequency (Salin et al., 1996), hence driving CA3 network activity very efficiently (Wiebe and Staubli, 1999; Henze et al., 2002). In addition, mossy fiber—CA3 synapses express presynaptic forms of long-term plasticity (LTP and LTD) that are expressed by persistent changes in the probability of glutamate release (Nicoll and Schmitz, 2005).

It is now emerging that many of these physiological processes are regulated by ionotropic receptors localized in presynaptic and perisynaptic membranes in mossy fibers themselves. In this paper, we discuss recent advances in our understanding of presynaptic receptor function at hippocampal mossy fiber synapses and expand on the view that they act as important modulators of synaptic transmission and plasticity in CA3 targets. We first introduce some of the techniques that have been employed to investigate presynaptic ionotropic receptors at mossy fibers. We then review the evidence showing the types of ionotropic receptors and the potential sources of neurotransmitters that can activate them, the downstream signaling mechanisms that ensue, and the differing forms of synaptic plasticity mediated by these receptors at synapses formed onto CA3 pyramidal neurons.

Several criteria must be met to unambiguously identify presynaptic ionotropic receptors in axon terminals. These include (1) ultrastructural localization of a particular receptor subunit to the presynaptic membrane, (2) detection of single channel activity in an excised patch from the presynaptic membrane; (3) evidence that exogenous activation of the receptor affects the presynaptic membrane potential or resistance. Moreover, (4) to argue that the receptor has a physiological role, it is necessary to show that it can be activated by the endogenous neurotransmitter upon activation of appropriate axons, and that this can be blocked or modulated by selective ligands.

## Methods available to investigate presynaptic receptor function

**Figure 1:**
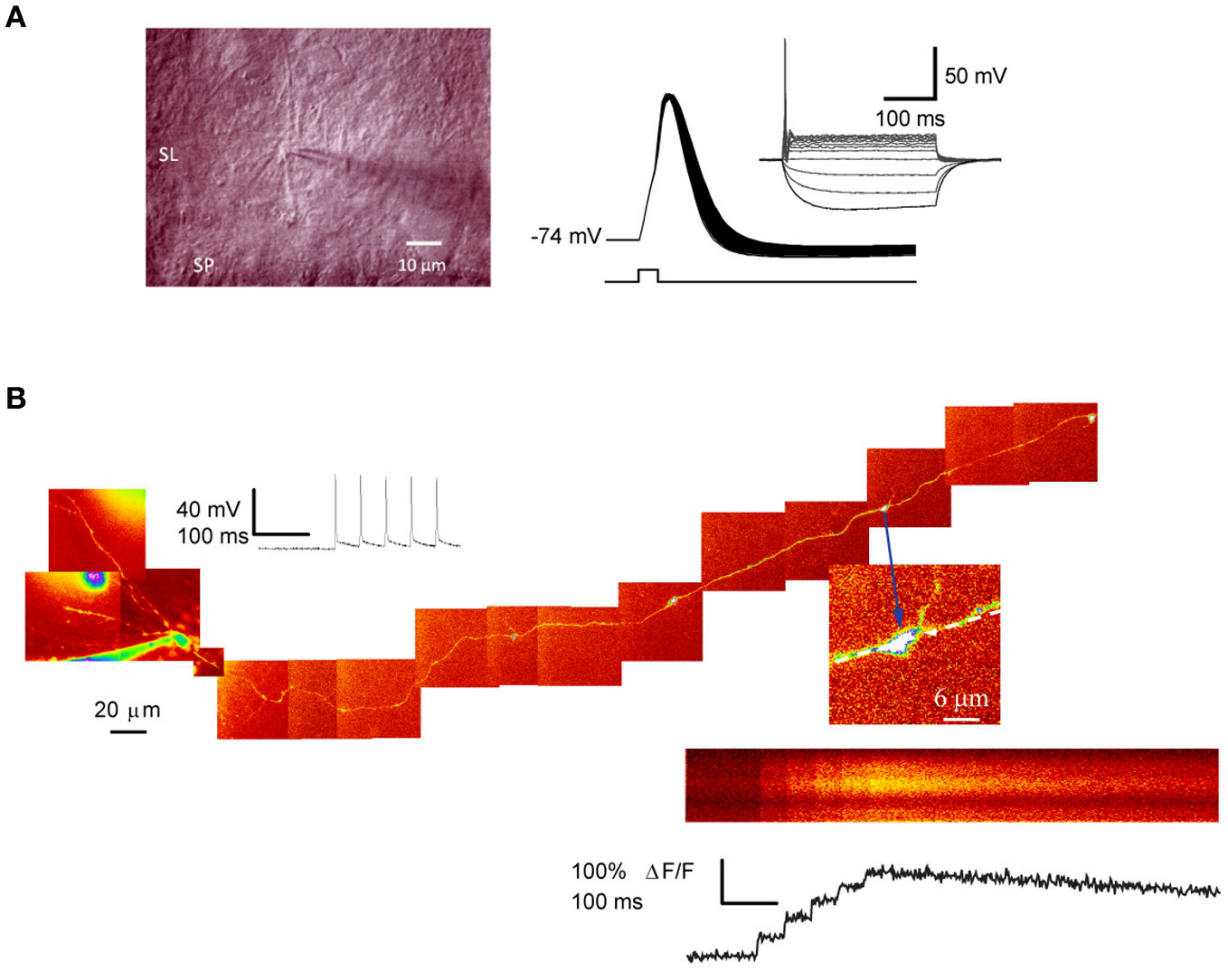
**Patch-clamp recording and 2-photon excitation fluorescence imaging from single giant mossy fiber boutons. (A)** Left, IR–DIC photomicrograph of a putative mossy fiber bouton in juxtaposition with a dendrite located in stratum-lucidum. Right: action potentials evoked by a single depolarizing current step (1 nA, 0.5 ms) repeated at 50 Hz. Note the spike broadening. Inset shows the I–V relation (−30 to 60 pA, 300 ms; 10 pA increment). **(B)** Ca^2+^imaging in a dentate granule cell filled with Alexa 594. The montage was obtained from Kalman-filtered averages of 10–15-μm stacks. Arrow indicates a giant mossy fiber bouton in stratum lucidum selected for imaging. Line-scan and fluorescence transients recorded from the same bouton, in response to five somatic action potentials. Panel **(A)** image and AP traces courtesy of Dr. D. Engel and panel **(B)** from Scott and Rusakov (2006).

Among the plethora of experimental approaches available for measuring changes in axonal excitability and release probability consecutive to receptor activation, only high-resolution imaging and presynaptic recordings allow direct access to the presynaptic compartment (Figure 1). All other methods that rely on electrophysiology and statistical analysis (miniature synaptic current frequency analysis, CV analysis and paired-pulse ratio of amplitude of electrically-evoked responses) can only yield indirect estimates and suffer serious drawbacks since the recordings are being made either from postsynaptic targets, the somatic compartment, or the extracellular space.

Briefly, when recording from postsynaptic cells, activation of the presynaptic receptor should affect the frequency of miniature synaptic currents in preference to their amplitudes, and the paired-pulse ratio of two consecutively evoked synaptic currents should be increased or decreased by presynaptic receptor activation (Zucker et al., 1998). However, using the paired-pulse ratio as a sole indicator of presynaptic receptor activation should be made with caution since postsynaptic mechanisms can also contribute (Kirischuk et al., 2002), and an autoreceptor function can also occlude the change in paired-pulse ratio expected of a presynaptic modulation. A complementary analysis is to compare the CV with the mean amplitude of evoked synaptic currents to deduce whether variation in synaptic efficacy has a presynaptic or postsynaptic locus. Generally, proportionate changes in CV^−2^ and mean amplitude imply presynaptic modulation of transmitter release, whereas changes to the mean amplitude without change in CV^−2^ imply modulation of postsynaptic receptors (Edwards et al., 1989; Manabe et al., 1993). However, further complications arise from this kind of analysis when considering other parameters that can influence baseline receptor occupancy and diffusion and uptake of neurotransmitters, such as the recording temperature (Rusakov and Kullmann, 1998; Perrais and Ropert, 1999).

Several other electrophysiological techniques can be deployed to investigate the effect of receptor activation on axonal excitability (Wall, 1958), the vast majority of which makes use of extracellular electrical stimulation combined with single axon unit recordings or recordings of the compound presynaptic action potential (the afferent volley). These methods often lack spatial resolution and can be sensitive to drift, hampering the analysis of the effect of receptor activation on single fiber threshold. They are also extremely sensitive to ionic shifts in the extracellular space, in particular to K^+^ that tends to accumulate during repetitive activity and can alter fiber threshold.

An alternative method to measure changes in fiber excitability is to record antidromic action potentials evoked via a stimulus electrode positioned in the axonal projection zone. In the voltage-clamp configuration, these are detected as action currents. Two approaches have been taken to examine changes in the probability for evoking antidromic action currents consecutive to receptor modulation. In its most simple implementation, a threshold-straddling stimulus is applied so that the success rate for evoking antidromic action currents is set to ~50%. Local application of drugs acting on axonal receptors should increase or decrease the success rate. A variant of the method is to cycle the stimulus through a saw-tooth intensity pattern ranging from 100% failures to 100% success for evoking an action current. Here, the effect of the drug on axonal receptors is examined over the entire stimulus—response relation. However, a recurrent problem with these approaches is that they rely on the integrity of axonal projections connected to their parent somata, a situation that occurs in about 12–15% of our slices.

An important breakthrough that allowed direct assessment was the development of direct patch-clamp recordings from large mossy fiber terminals (Geiger and Jonas, 2000; Bischofberger et al., 2006), thus enabling a detailed characterization of the pharmacological and biophysical properties of presynaptic receptors at single channel level. Although these recordings have provided important insights into the relation between receptor activation and presynaptic membrane potential, they inevitably perturb intraterminal ionic gradients because the size of the bouton is very small in relation to the patch pipette. This is particularly problematic when measuring the membrane potential from whole-terminal recordings because this method clamps [K^+^]_i_ and because the input resistance of the clamped structure is comparable to the seal resistance (Verheugen et al., 1999; Tyzio et al., 2003). It also affects the [Cl^−^]_i_ which has been estimated ~20 mM in the Calyx of Held (Price and Trussell, 2006), therefore potentially compromising the driving force for GABA_A_ receptor and glycine receptor mediated ion fluxes.

The membrane potential of mossy fiber boutons can in principle be estimated non-invasively (Fricker et al., 1999; Verheugen et al., 1999). When recording in bouton-attached mode with a high [K^+^] pipette solution and applying a voltage ramp (from a holding potential of −100 to +200 mV), a K^+^ conductance is activated such that the K^+^ current reverses when the pipette potential is equal to the transmembrane potential. There are several shortcomings of this bouton-attached configuration mainly related to the small size of the patched structure: first, the [K^+^]_i_ in the terminal is unknown and is assumed to be as high as in the somatic compartment, but this potential source of error has only a minor influence (Fricker et al., 1999). Second, a major bias occurs when the seal resistance is equivalent or lower than the resistance of the patch, in which case, most of the K^+^ current will be shunt (It should not however influence the potential at which the current changes polarity). That said, gramicidin perforated-patch recordings, which normally do not perturb [Cl^−^]_i_ have not been successfully applied to mossy fiber boutons.

Large mossy fiber varicosities can be visualized in living slices with two-photon excitation fluorescence imaging integrated with patch-clamp electrophysiology (Scott and Rusakov, 2006, 2008; Nistico et al., 2012). Granule cell loading with a morphological tracer such as Alexa Fluor 594 (20–40 μM), together with a high-affinity Ca^2+^ indicator, Fluo-4 (200 μM), or Oregon Green BAPTA-1 (200 μM), then potentially allows Ca^2+^ signaling in unambiguously identified mossy fiber boutons. This depends on tracing the axon from the soma through the hilus and into stratum lucidum. The success rate for imaging a giant mossy fiber bouton in CA3 supplied by a given granule cell is however low, because it depends on the integrity of a lengthy mossy fiber connected to its parent soma.

Presynaptic ionotropic receptors can also been investigated in acutely dissociated CA3 pyramidal neurons, which can be isolated with adherent functional synaptic terminals. This technique (Akaike and Moorhouse, 2003) offers the advantage that single presynaptic terminals and boutons can be visualized using fluorescent markers and can be focally stimulated with a glass micropipette. Adherent contacts are functional and generate spontaneous postsynaptic currents over a reasonable period of time, thus enabling pharmacological manipulation of presynaptic receptors. The method has been successfully applied to CA3 pyramidal neurons (Jang et al., 2006). However, it is mainly restricted to proximal contacts since the dissociation procedure eliminates most of dendritic processes in postsynaptic neurons.

## Presynaptic GABA_A_ receptors

Modulation of transmitter release at a synapse was first demonstrated in the pioneering studies of Dudel and Kuffler (1961) and Eccles (1964) who showed that presynaptic GABA receptors inhibited transmitter release from crustacean motor neuron terminals and vertebrate sensory neuron terminals in the spinal cord, respectively. Since then, presynaptic GABA_A_ receptors have been described in the retina (Tachibana and Kaneko, 1984; Lukasiewicz and Werblin, 1994), the cerebellum (Trigo et al., 2007), the posterior pituitary (Zhang and Jackson, 1993), thalamic nuclei (Jang et al., 2001), and higher cortical structures where they have been shown to modulate axonal excitability and the release of neurotransmitters (Kullmann et al., 2005).

GABA_A_ receptors depolarize presynaptic axons because [Cl^−^]_i_ is relatively high, reflecting absence of the main extrusion system KCC2 (Gulyas et al., 2001; Hubner et al., 2001). Opening of GABA_A_ receptors may interfere with the propagation of action potentials by decreasing membrane resistivity (Segev, 1990; Cattaert and El Manira, 1999; Wachowiak and Cohen, 1999; Verdier et al., 2003; Alle and Geiger, 2007). Others have argued that GABA_A_ receptor-mediated depolarization could decrease the driving force for Ca^2+^ and/or inactivate Na^+^ channels (Graham and Redman, 1994). However, presynaptic depolarization consecutive to GABA_A_ receptor activation enhances neurotransmitter release at the MNTB synapse in the auditory brainstem (Turecek and Trussell, 2001, 2002). This effect appears to be mediated by an increase in basal Ca^2+^ (Awatramani et al., 2005), and recent evidence suggests that P/Q-type Ca^2+^ channels can be enhanced in a [Ca^2+^]_i_-dependent manner (Ishikawa et al., 2005; Hori and Takahashi, 2009).

**Figure 2:**
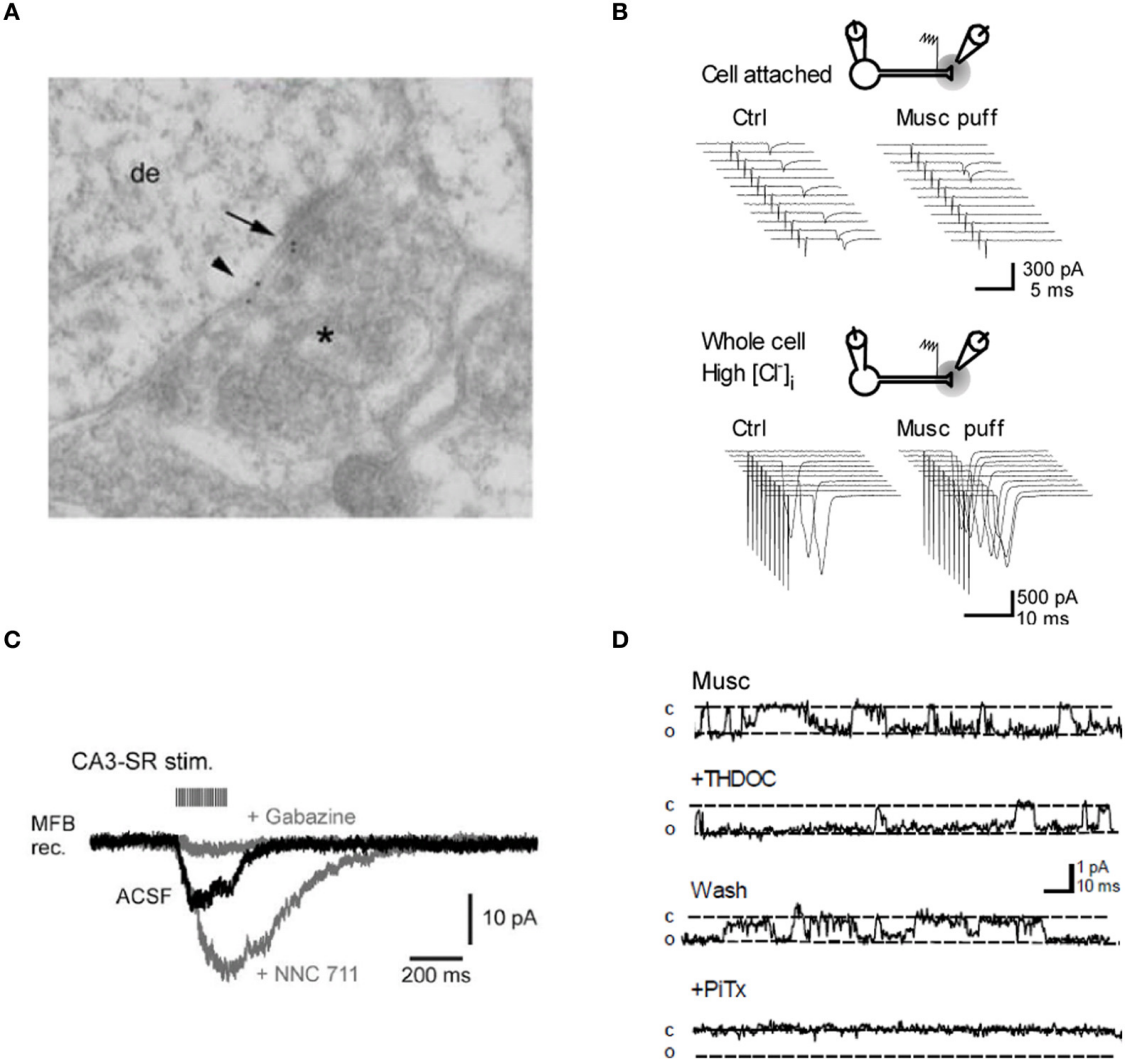
**Evidence for GABA_A_ receptors modulating mossy fiber excitability. (A)** Post-embedding immunolocalization of the GABA_A_ receptor subunit α_2_ in a presumptive large mossy fiber terminal. (Asterisk indicates the presynaptic profile). Labeling is present at the synaptic cleft (arrow) and at extrasynaptic axonal membranes (small arrowhead). **(B)** Top, intermittent antidromic action currents. Local pressure application of the GABA_A_ agonist muscimol close to the stimulation site decreased the axon excitability. Bottom, following “break-in” with a pipette containing a high [Cl^−^], the same application of muscimol caused an increase in axon excitability. Sweeps on the right are from one example cell. **(C)** Spill-over GABA_A_ receptor-mediated currents in a mossy fiber bouton held in voltage-clamp. Twenty pulses at 100 Hz applied to stratum radiatum in area CA3. Gray traces, successive bath application of the GAT 1 blocker NNC 711 (1 μM) and the GABA_A_ receptor antagonist gabazine (10 μM). **(D)** Muscimol-activated single channel currents in an outside-out patch from a mossy fiber bouton recorded with symmetrical [Cl^−^]. Superfusion of the neurosteroid THDOC (10 nM) reversibly prolongs the apparent open probability of the channel whereas the GABA_A_ receptor antagonist picrotoxine blocks the current. Panels **(A, B,** and **D)** were reproduced from Ruiz et al. (2003, 2010) and panel **(C)** from Alle and Geiger (2007).

Nearly a decade ago, we provided the first demonstration that presynaptic GABA_A_ receptors occur in axon terminals in the hippocampus (Ruiz et al., 2003). We obtained both ultrastructural and pharmacological evidence consistent with the presence of presynaptic GABA_A_ receptors containing α_2_ (Figure 2A) subunits (see also Jang et al., 2006; Alle and Geiger, 2007). By altering [Cl^−^]_i_ within individual granule cells we showed that the GABA_A_ receptor agonist muscimol produced opposite changes in the probability for evoking antidromic action potentials (Figure 2B). Opposite effects on axonal excitability were also obtained by applying the GABA_A_ receptor blocker SR95531 (gabazine) depending on [Cl^−^]_i_, suggesting that these receptors are tonically active. These results could only be explained by the presence of GABA_A_ receptors on mossy fibers. Measurements of single channel openings of presynaptic GABA_A_ receptors in outside-out patches from mossy fiber boutons yielded an estimate 36 pS (Alle and Geiger, 2007), similar to the main conductance state of GABA_A_ receptors found in other preparations (Jones and Westbrook, 1995; Turecek and Trussell, 2002), but slightly larger than our own measurements (24 pS; Ruiz et al., 2010). Their expression is also developmentally regulated. Nakamura et al. (2007) showed that mossy fiber GABA_A_ receptors are involved in the activity-dependent facilitation of the fiber volley via depolarizing GABA actions, a phenomenon that gradually decreased with development and eventually vanished at around postnatal day 30. In contrast, Alle and Geiger (2007) found that functional presynaptic GABA_A_ receptors are conserved during development as witnessed by the presence of GABA_A_ receptor-mediated currents in mossy fiber boutons from 3 month old rats.

What is the normal mode of activation of these receptors? We demonstrated that mossy fiber excitability was modulated by trains of stimuli designed to release GABA from neighboring inhibitory synapses, implying that presynaptic GABA_A_ receptors could be activated by GABA spillover (Ruiz et al., 2003). This finding was later confirmed by Alle and Geiger (2007) who characterized a slow and small amplitude current in mossy fiber boutons, in response to stimulus trains (Figure 2C). These spillover currents were abolished by application of the GABA_A_ receptor antagonist gabazine, whereas blocking the main GABA uptake system GAT-1 with NNC-711 enhanced them. The results argued that presynaptic GABA_A_ receptors can detect activity-dependent fluctuations in the extracellular GABA concentration (see also Nakamura et al., 2007) as shown for a form of GABA_B_ receptor-mediated signaling at these terminals (Vogt and Nicoll, 1999; Chandler et al., 2003; Safiulina and Cherubini, 2009).

Dentate granule cells express tonically active GABA_A_ receptors, which are sensitive to physiological concentrations of tetrahydrodeoxycorticosterone (THDOC), an endogenous neurosteroid that is relatively selective for δ-subunit containing receptors (Stell et al., 2003). We found that 10 nM THDOC reversibly reduces the excitability of mossy fibers, mimicking the effect of GABA_A_ receptor agonists and suggesting that high-affinity δ-subunit containing receptors (in addition to α_2_) are present in the axon. Similar results were obtained with the hypnotic compound THIP (gaboxadol, 100 nM), which is a relatively selective agonist at GABA_A_ receptors that lack γ subunits. Finally, we confirmed that THDOC increased the apparent open probability of GABA_A_ receptors in outside-out patches from mossy fiber boutons but had no effect on the single-channel conductance (Figure 2D).

**Figure 3:**
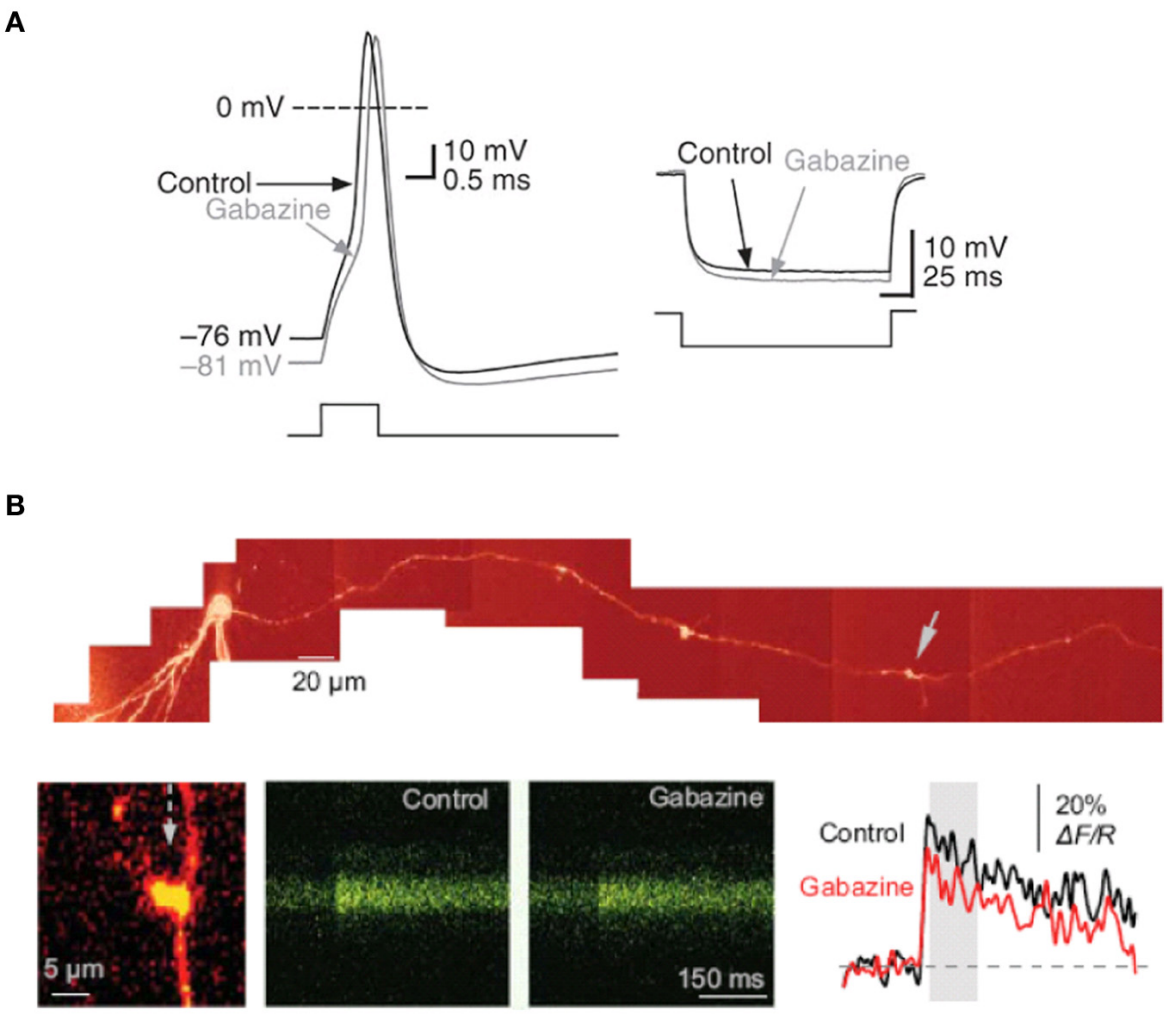
**Tonically-active GABA_A_ receptor–mediated currents modulate the electrical properties of mossy fiber boutons. (A)** Current-clamp recording from a mossy fiber bouton with 20 mM [Cl^−^]_i_. Left, sample traces showing the action potential before (black) and after (gray) superfusion of gabazine (5 μM). Right, response to hyperpolarizing current injection showing an increase in mossy fiber input resistance in gabazine. **(B)** Tonically-active GABA_A_ receptors enhance presynaptic action potential-dependent Ca^2+^ transients in giant mossy fiber boutons. Top, reconstruction of a dentate granule cell (Alexa Fluor 594 channel, λ x = 800 nm). Arrows indicate giant boutons with characteristic thin filopodia. Bottom, blocking GABA_A_ receptors with gabazine reduced spike-dependent presynaptic Ca^2+^ entry. Line scans and traces are Ca^2+^ responses in the mossy fiber bouton shown on the left following a single action potential induced at the soma, in control conditions and in 1 μM gabazine. Reproduced from Ruiz et al. (2010).

We further showed that tonically-active presynaptic GABA_A_ receptors depolarize mossy fibers and modulate the input resistance of mossy fiber boutons, as well as the shape of presynaptic action potentials (Figure 3A). Blocking GABA_A_ receptors with gabazine hyperpolarized mossy fiber boutons and reduced spike half-width, whereas THDOC had the opposite depolarizing effect and broadened action potentials. These results diverged from those of Alle and Geiger (2007) who did not detect a change in mossy fiber bouton input resistance or membrane potential as a consequence of GABA_A_ receptor blockade. The reason for this discrepancy is unclear but it could involve differences in rat strains (Wistar vs. Sprague-Dawley), recording temperature and state of neuromodulation, for example by zinc or dynorphin. Notwithstanding these differences, the presence of both high- and low-affinity presynaptic GABA_A_ receptors suggests a richness of phasic and tonic modulation of synaptic transmission at mossy fiber—CA3 synapses.

**Figure 4:**
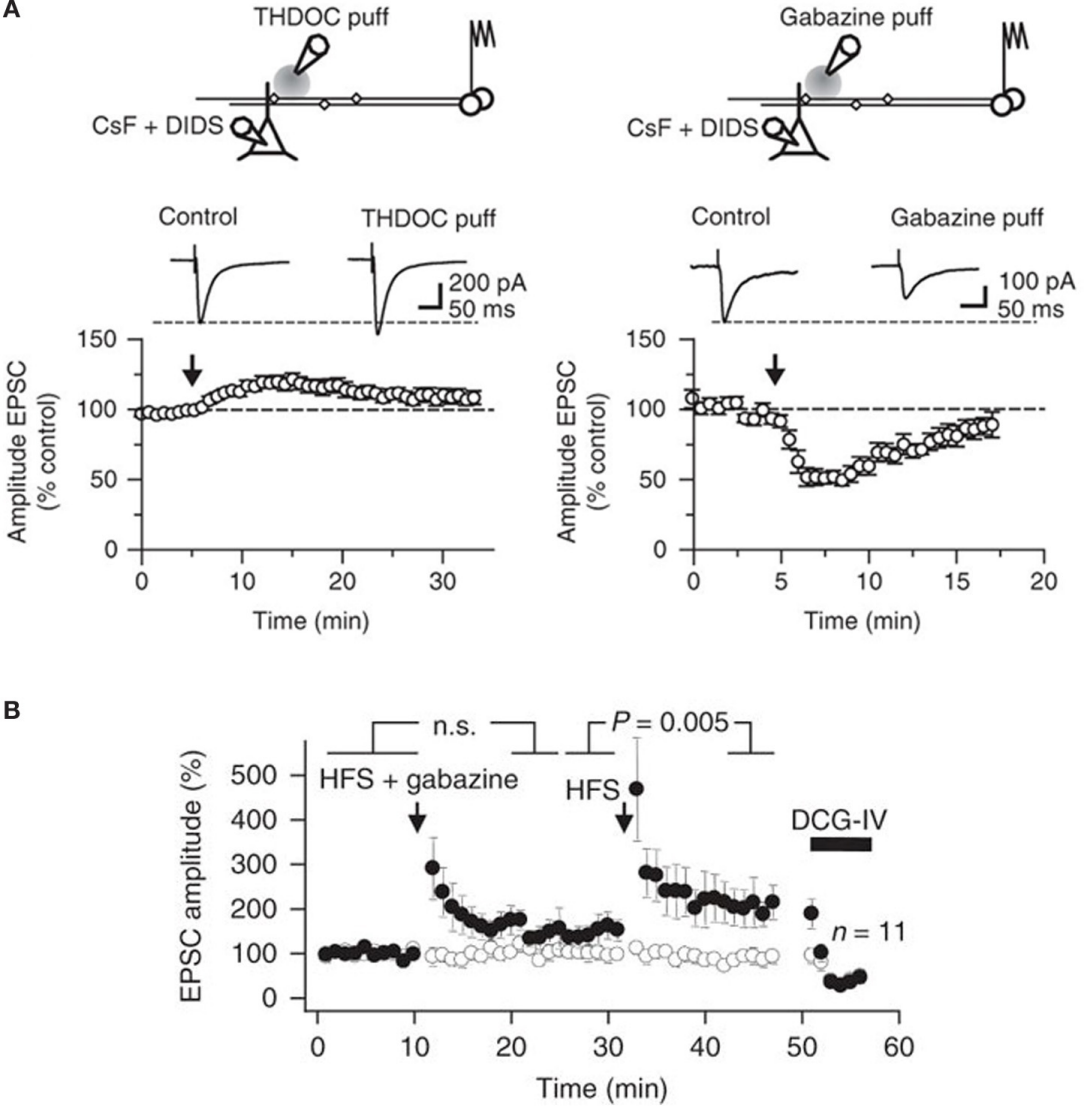
**Bidirectional modulation of synaptic transmission via tonically active GABA_A_ receptors at mossy fibers. (A)** Top, schematic illustrating the experimental design used to study presynaptic GABA_A_ receptors at mossy fiber—CA3 synapses. Postsynaptic GABA_A_ receptors in CA3 pyramidal cells were blocked using an intracellular pipette solution containing CsF and DIDS. A pressure-application pipette containing THDOC (50 nM, left) was positioned ~50–200 μm from the apical dendrite of the recorded neurons. Bottom, time course of the effects of pressure application of THDOC (left; *n* = 8). Traces are averages of 10 consecutive EPSCs before and after drug application. The effect of gabazine (10 μM) applied as in **(A)** is also shown (right; *n* = 6). **(B)** EPSC amplitudes recorded in a CA3 pyramidal neuron in response to stimulation of two pathways. High frequency stimulation (HFS) was delivered to one pathway (filled symbols) at the times indicated, either together with local pressure application of gabazine in stratum lucidum (HFS + gabazine) or alone (HFS). DCG-IV was applied at the end of the experiment to confirm that the responses were profoundly depressed, typical of mossy fibers. Average time course of 11 experiments (error bars indicate s.e.m). Reproduced from Ruiz et al. (2010).

We also detected a strong influence of GABA_A_ receptors on action-potential dependent Ca^2+^ transients in single axonal varicosities imaged with 2-photon excitation fluorescence microscopy (Figure 3B). We showed that the GABA_A_ receptor antagonist gabazine decreased the amplitude of action-potential evoked Ca^2+^ transients whereas THDOC had the opposite effect. These results demonstrated that tonically-active GABA_A_ receptors contribute to presynaptic depolarization and Ca^2+^ influx when a single action potential invades a mossy fiber varicosity. To assess the contribution of presynaptic GABA_A_ receptors to glutamatergic transmission we recorded from CA3 pyramidal neurons with a pipette solution containing CsF and 4,4'-diisothiocyanostilbene-2,2'-disulfonic acid (DIDS) to block postsynaptic GABA_A_ receptors intracellularly, while leaving presynaptic receptors unaffected (Figure 4A). Local pressure application of THDOC reversibly increased the amplitude of evoked excitatory postsynaptic currents (EPSCs) whereas gabazine decreased it, implying that presynaptic GABA_A_ receptor normally exert a bidirectional control over dentate gyrus—CA3 neurotransmission. Finally, we demonstrated that blocking presynaptic GABA_A_ receptors impaired the induction of mossy fiber LTP (Figure 4B). A straightforward explanation for this finding was that tonic GABA_A_ receptor mediated presynaptic depolarization has a permissive role in mossy fiber LTP because its induction is steeply dependent on the presynaptic membrane potential and Ca^2+^ influx (Castillo et al., 1994; Schmitz et al., 2003; Nicoll and Schmitz, 2005). Although the evidence for presynaptic GABA_A_ receptors is compelling some puzzles remain. For instance, muscimol decreases mossy fiber excitability even though GABA_A_ receptor activation depolarizes boutons (Alle and Geiger, 2007; Ruiz et al., 2010). Reduced excitability can potentially be explained by partial inactivation of Na^+^ channels. Furthermore, although differences in apparent affinity of GABA_A_ receptors suggest that multiple biophysically distinct receptors coexist in the same boutons they have not been resolved at single channel resolution.

## Presynaptic glycine receptors

Glycine is a common neurotransmitter in the spinal cord and the brainstem whose action on glycine receptors activates a Cl^−^ conductance. Glycine and glycine receptors are, however, not restricted to these regions, nor do they always promote inhibition. Glycine receptors have been reported in the cerebellum and the hippocampus (Araki et al., 1988). Glycine receptors in the forebrain are mainly found at extrasynaptic sites in postsynaptic membranes or even presynaptically (Harvey and Rigo, 2010). In the retina, presynaptic glycine receptors localize in rod cells terminals where they inhibit transmission by hyperpolarization and a shunting mechanism (Morkve and Hartveit, 2009). Presynaptic glycine receptors enhance glutamate release at the MNTB synapse by promoting small depolarization's of the nerve terminal (Turecek and Trussell, 2001). Interestingly, presynaptic glycine receptor activation in the ventral tegmental area enhances GABA release in young neurons but has an inhibitory effect in older neurons (Ye et al., 2004). Thus, the direction in which transmitter release is regulated by presynaptic glycine receptors varies within the CNS and during development, and this can affect both excitatory and inhibitory neurotransmitters.

**Figure 5:**
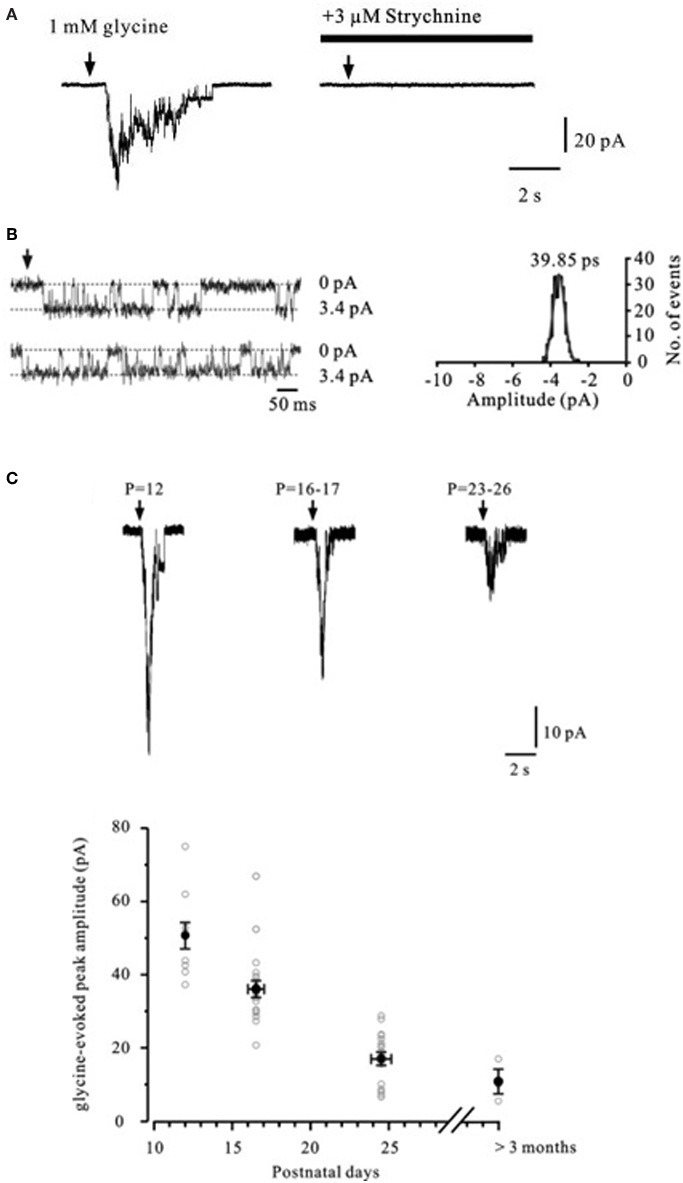
**Glycine-evoked currents in mossy fiber boutons. (A)** Currents in a mossy fiber bouton evoked by focal application of 1 mM glycine. Superfusion of the glycine receptor antagonist strychnine (3 μM) completely blocks the current. Arrows indicate glycine puff-application. **(B)** Left, representative traces of single-channel currents in an isolated outside-out patch from a mossy fiber bouton (P12) evoked by continuous application of 1 mM glycine (symmetrical [Cl^−^]). Dashed lines indicate either baseline current or glycine induced inward current during channel opening. Right, Gaussian fit to the single-channel current amplitude histogram indicates a single-channel conductance of 40 pS. **(C)** Currents evoked by 1 mM glycine recorded from mossy fiber boutons in different age groups. Reproduced from Kubota et al. (2010).

In the hippocampus, the evidence for functional presynaptic glycine receptors is sparse. Exogenously applied glycine significantly increased the frequency of spontaneous EPSCs recorded from mechanically dissociated rat dentate hilar neurons attached with native presynaptic nerve terminals (Lee et al., 2009). The enhancing effect of glycine on synaptic transmission was blocked by the specific glycine receptor antagonist strychnine, but was unaffected by picrotoxin. The evidence that these receptors are expressed at mossy fibers again comes from direct patch-clamp recordings and single channel analysis (Kubota et al., 2010). As shown in Figure 5A, immature mossy fiber boutons challenged with glycine displayed inward currents that were blocked by strychnine. Glycine-gated channels showed a main conductance of 40 pS (Figure 5B) but multiple conductance states were observed, consistent with expression of both homo- and hetero-oligomeric glycine receptors (Takahashi and Momiyama, 1991; Singer and Berger, 1999). The expression profile of presynaptic glycine receptors at mossy fibers declined dramatically with age (Figure 5C) in sharp contrast with those found at the Calyx of Held (Turecek and Trussell, 2001, 2002).

## Presynaptic NMDA receptors

One of the first demonstration that presynaptic NMDA receptors occur on axon terminals in the CNS came from the immunolocalization of the NMDA receptor subunit NR1 in both the dorsal and ventral horns of the rat spinal cord, particularly near the active zones, suggesting a modulatory role in transmitter release (Liu et al., 1994). These receptors were later found to modulate the release of glutamate and substance P from nociceptive fibers (Liu et al., 1997) and glutamate release from primary sensory neurons (Bardoni et al., 2004). In the cerebellum, presynaptic NMDA receptors facilitate GABA release at basket cell—Purkinje cell synapses via retrograde signaling and release of Ca^2+^ from internal stores (Duguid and Smart, 2004). In cortical regions, Berretta and Jones (1996) introduced a trick to specifically block NMDA receptors in postsynaptic cells, leaving presynaptic NMDA receptors available for pharmacological manipulation. They recorded in whole-cell mode from layer II enthorinal cortex neurons with a pipette solution containing 1 mM MK-801 to block NMDA receptors intracellularly. They showed that superfusion of the NMDA receptor antagonist D-AP5 resulted in a decrease of the frequency of miniature EPSCs with little effect on the amplitude, suggesting that tonically-activated NMDA receptors facilitate glutamate release also at cortical synapses. Finally, in the juvenile rat barrel cortex, presynaptic NR2B-containing NMDA receptors enhance AMPA receptor-mediated synaptic transmission at layer 4 to layers 2/3 synapses (Brasier and Feldman, 2008).

Recently, presynaptic NMDA receptors have been implicated in spike-timing dependent long-term depression at neocortical synapses (Sjostrom et al., 2003; Buchanan et al., 2012). This process requires the co-activation of CB1 receptors and is developmentally regulated such that in the juvenile, the NR3A subunit enhances spontaneous and evoked glutamate release and is required for spike timing-dependent long-term depression, whereas in the adult, NR2B-containing presynaptic NMDA receptors enhance neurotransmission in the absence of Mg^2+^, implying that they function under depolarizing conditions. Recently, a new caged compound has demonstrated axonal NMDA receptors required for induction and the presynaptic locus of expression of LTD at layer 4-layer 2/3 synapses (Rodriguez-Moreno et al., 2012). The evidence for presynaptic NMDA receptors in cortical neurons is however incomplete, and one study failed to detect them using axonal Ca^2+^ imaging (Pugh and Jahr, 2012).

Evidence for presynaptic NMDA receptors at mossy fiber synapses is also incomplete. In the monkey hippocampus, monoclonal antibodies raised against the NMDA receptor subunits NR1 and NR2 stained stratum lucidum but not postsynaptic targets (Siegel et al., 1994). The detection of NMDA receptors subunits in the mossy fiber projection zone does not, however, prove the presence of functionally active presynaptic receptors. A study reporting NMDA receptors using patch-clamp recordings from mossy fiber boutons has only been reported in abstract form (Alle and Geiger, 2005). Thus, evidence that NMDA receptors are expressed in mossy fiber terminals is far from conclusive.

## Presynaptic kainate receptors

In contrast with AMPA and NMDA receptors, kainate receptors play a relatively small role in fast glutamatergic transmission at most synapses. Some exceptions are mossy fiber synapses, and thalamo-cortical synapses early in development, where they mediate slow and small amplitude EPSCs (Castillo et al., 1997; Vignes and Collingridge, 1997; Kidd et al., 2002). There is however, abundant evidence for kainate receptor expression in presynaptic terminals where they modulate the plastic properties of both excitatory and inhibitory connections (reviewed by Kullmann, 2001; Pinheiro and Mulle, 2008; Contractor et al., 2012). In the hippocampus, early binding studies using (3)H-radiolabelled kainate as ligand (Foster et al., 1981; Monaghan and Cotman, 1982; Bahn et al., 1994) have shown the presence of high-affinity binding sites restricted to stratum lucidum, where mossy fibers terminate. This staining pattern was dramatically reduced by selective destruction of dentate granule cells with the antimitotic agent colchicine (Represa et al., 1987), suggesting that kainate receptors are expressed in the axon or in presynaptic terminals. Immunohistochemical experiments with monoclonal antibodies directed against GluK1 and GluK2 also stained stratum lucidum (Petralia et al., 1994), in line with previous results.

**Figure 6:**
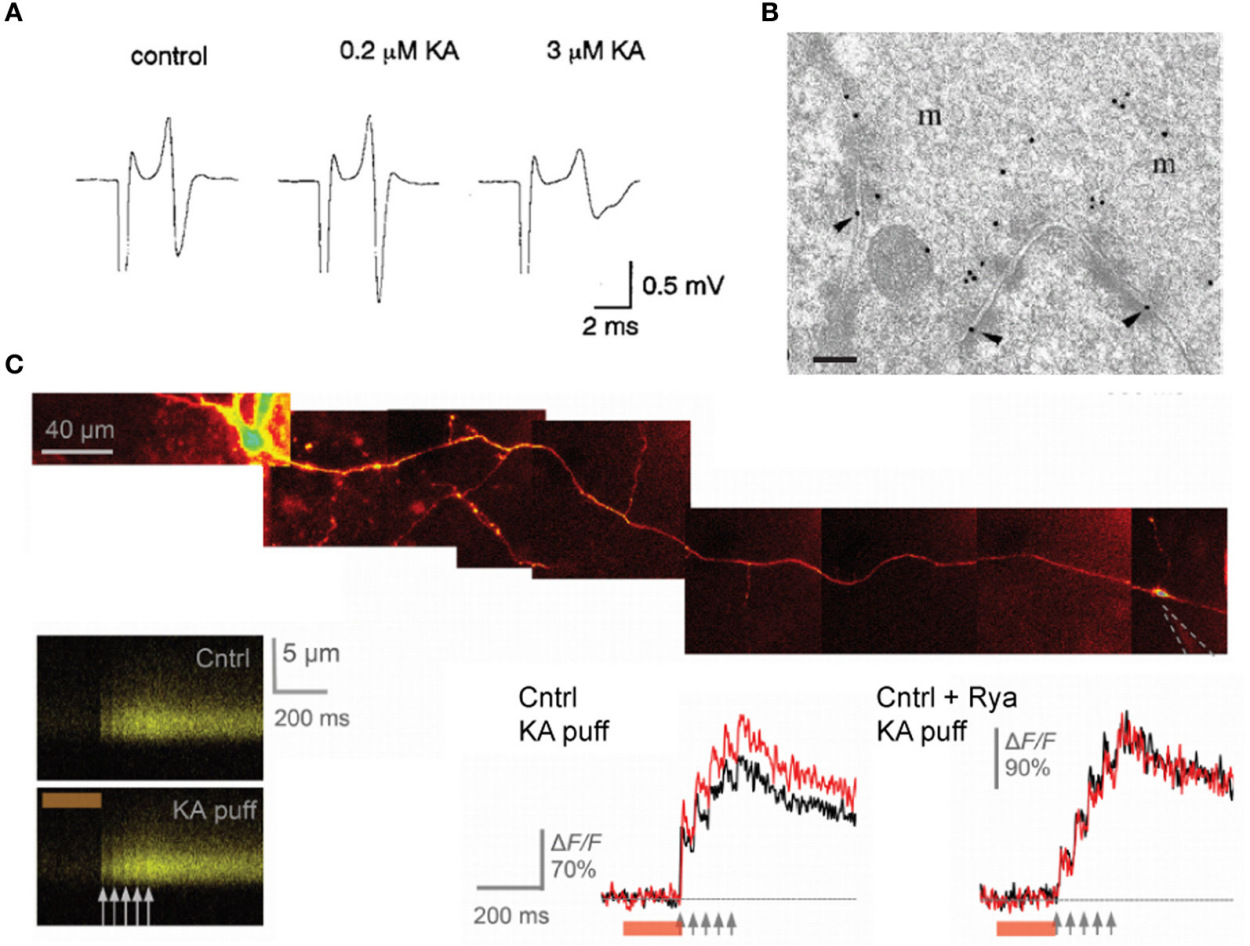
**presynaptic kainate receptors modulate mossy fiber excitability and Ca^2+^ influx in single varicosities. (A)** Representative traces of the afferent volley before and during superfusion of low and high doses of kainate, as indicated. **(B)** High-magnification micrograph of stratum lucidum showing a mossy fiber terminal labeled with antibody fragments directed against GluK4. Immunogold particles are found along the presynaptic membrane. Scale bar, 20 nm. **(C)** Local kainate application mobilizes Ca^2+^-store and boosts action-potential-evoked Ca^2+^entry in giant mossy fiber boutons. Top, a granule cell axon is traced from the soma and a patch pipette (dotted lines) is approached near a giant mossy fiber bouton. Kainate is pressure ejected together with Alexa Fluor 594 from the pipette using a 200 ms pressure pulse (line-scans). Local pressure application of kainate has no effect on the baseline resting Ca^2+^ but increases action-potential-evoked Ca^2+^ entry in the imaged bouton. Ca^2+^ store depletion with ryanodine abolishes the presynaptic effects of kainate application. Panel **(A)** is reproduced from Kamiya and Ozawa (2000), panel **(B)** from Darstein et al. (2003) and panel **(C)** from Scott et al. (2008).

The effects of kainate on dentate gyrus—CA3 neurotransmission are concentration-dependent and bimodal: superfusion of a low dose of kainate (50–200 nM) has been reported to facilitate evoked EPSC recorded in CA3 pyramidal neurons whereas higher doses (1–5 μM) have a depressant effect. The facilitation of mossy fiber EPSCs consecutive to the application of a low concentration of kainate is accompanied by an enhancement of the presynaptic fiber volley (Figure 6A; Kamiya and Ozawa, 2000; Schmitz et al., 2001) and by a decrease in the threshold for evoking antidromic action potentials recorded in granule cells (Kamiya and Ozawa, 2000), consistent with a presynaptic expression of kainate receptors. One explanation for these effects is that mild depolarization could inactivate K^+^ channels whereas inactivation of presynaptic Ca^2+^ channels may occur with large doses of kainate. Presynaptic kainate eceptors could be activated by synaptic release of glutamate from neighboring mossy fibers or associational-commisural fibers, thus mimicking the effect of exogenous agonist application on the fiber volley and threshold (Schmitz et al., 2000).

Although kainate receptors contribute to the large frequency-dependent short-term plasticity of mossy fiber synapses, the identity of the subunits that mediate these effects is unclear. Studies performed in knock-out mice indicate that presynaptic kainate receptors containing the GluK2 (Contractor et al., 2001; Breustedt and Schmitz, 2004; Rodriguez-Moreno and Sihra, 2007) and GluK3 subunits (Pinheiro et al., 2007; Perrais et al., 2009) facilitate the induction of this form of synaptic plasticity whereas other studies have identified GluK1 as a principal player (Bortolotto et al., 1999; Lauri et al., 2001; More et al., 2004). The reasons for these discrepancies are unknown, but some of the GluK1 antagonists used in these studies could also block receptors containing the GluK3 subunit (Perrais et al., 2009). GluK3 is thought to underlie the effects mediated by presynaptic kainate receptors. Receptors containing this subunit have a low sensitivity for glutamate, are highly Ca^2+^-permeable, desensitize rapidly and are subject to a voltage-dependent block by intracellular spermine. However, the view that kainate receptors participate in short-term plasticity at mossy fiber—CA3 synapses was recently challenged by Kwon and Castillo (2008). The authors found no evidence that presynaptic kainate receptors facilitate transmitter release on CA3 pyramidal neurons over a wide range of stimulus frequencies delivered to mossy fibers, and argued that actions generally attributed to presynaptic kainate receptors are likely due to activation of the recurrent CA3 network.

Two studies have provided solid and direct evidence that these receptors actually occur in presynaptic and perisynaptic membranes in mossy fibers. The first study is the immunolocalization of kainate receptor subunits at ultrastructural level performed by Darstein et al. (2003). The authors showed that both GluK4 and GluK5 subunits localize presynaptically, with a preferential expression for GluK4, whereas GluK5 is mainly found in postsynaptic membranes (Figure 6B). Interestingly, antibodies against these two subunits also pulled-down GluK2 in hippocampal membrane extracts but failed to detect GluK3 subunits, whose localization is thought to be presynaptic (Pinheiro et al., 2007). The second report by Scott et al. (2008) used high resolution multi-photon imaging and pharmacology. Here the authors showed that individual action potentials evoke an increase in intracellular Ca^2+^ in presynaptic varicosities which is enhanced by kainate receptor activation, and contribute to activity-dependent facilitation of synaptic transmission in CA3 pyramidal neurons (Figure 6C). Whether glutamate released from a single varicosity acts on kainate receptors located on that same varicosity remains, however, to be demonstrated.

Kainate receptors are also thought to facilitate the induction of long-term potentiation in CA3 pyramidal neurons (Bortolotto et al., 1999; Lauri et al., 2001; Schmitz et al., 2003; Pinheiro et al., 2007). Again, conflictive reports have emerged in the literature about their role in the phenomenon (Kullmann, 2001). Recently, it has been argued that differences in kainate receptor involvement in mossy fiber long-term potentiation depend on slice orientation (Sherwood et al., 2012). In transverse slices, LTP was found resistant to GluK1 antagonists whereas in parasagittal slices LTP was consistently blocked by these agents. Whatever the explanations, we believe that many answers will come from direct recordings from mossy fiber boutons and a detailed characterization of the effects of kainate on the presynaptic membrane potential.

## Functional implications

Presynaptic modulation via ionotropic receptors may have broader implications for information processing and hippocampal physiology than previously thought. Endogenous levels of glutamate, GABA or glycine in brain tissue seem sufficient to promote baseline activity of high-affinity receptors, whereas receptors with lower affinities might be activated only after sustained activity and during a short time window consecutive to neurotransmitter release. For example, presynaptic kainate receptors at thalamo-cortical synapses depress glutamate release during repetitive activation at frequencies >50 Hz (Kidd et al., 2002), but presynaptic GABA_A_ receptors at mossy fibers regulate glutamate release when presynaptic activity spans a wider range of frequencies (Nakamura et al., 2007). Presynaptic ionotropic receptors are also subject to neuromodulation from a large variety of hormones, cations, and other neurotransmitters, including monoamines and neurosteroids, whose levels fluctuate extensively in physiological states and behavioral tasks as well as in pathological conditions. Finally, the permissive and synergistic effect of presynaptic GABA_A_ and kainate receptors on mossy fiber LTP highlight a powerful mechanism for information storage in CA3 networks. It also serves the basis for homeostatic regulation of feed-forward and frequency-tuned inhibition at a major input to the hippocampus proper.

## Conclusions

An increasing diversity of receptors normally found in dendrites are also localized in axon terminals where they mediate fast and local regulation of presynaptic excitability, Ca^2+^ influx and neurotransmitter release. A common feature of these receptors is that their activation leads to membrane depolarization and shunting, which in turn alter spike shape and the relation between Ca^2+^ influx and release probability. Direct recordings from mossy fiber boutons have provided some of the most compelling insights into the identity, localization, and physiological roles of these receptors.

### Conflict of interest statement

The authors declare that the research was conducted in the absence of any commercial or financial relationships that could be construed as a potential conflict of interest.
